# High *WT1* expression is an early predictor for relapse in patients with acute promyelocytic leukemia in first remission with negative *PML-RARa* after anthracycline-based chemotherapy: a single-center cohort study

**DOI:** 10.1186/s13045-017-0404-4

**Published:** 2017-01-23

**Authors:** Jae-Ho Yoon, Hee-Je Kim, Dae-Hun Kwak, Sung-Soo Park, Young-Woo Jeon, Sung-Eun Lee, Byung-Sik Cho, Ki-Seong Eom, Yoo-Jin Kim, Seok Lee, Chang-Ki Min, Seok-Goo Cho, Dong-Wook Kim, Jong Wook Lee, Woo-Sung Min

**Affiliations:** Department of Hematology, Catholic Blood and Marrow Transplantation Center, Leukemia Research Institute, Seoul St. Mary’s Hospital, College of Medicine, The Catholic University of Korea, 222 Banpodaero, Seocho-gu, Seoul, 06591 Korea

**Keywords:** Acute promyelocytic leukemia, *WT1*, *FLT3* mutation, Minimal residual disease

## Abstract

**Abstract:**

Wilms’ tumor gene 1 (*WT1*) expression is a well-known predictor for relapse in acute myeloid leukemia. We monitored *WT1* decrement along the treatment course to identify its significant role as a marker for residual disease in acute promyelocytic leukemia (APL) and tried to suggest its significance for relapse prediction. In this single center retrospective study, we serially measured *PML-RARa* and *WT1* expression from 117 APL patients at diagnosis, at post-induction and post-consolidation chemotherapies, and at every 3 months after starting maintenance therapy. All 117 patients were in molecular remission after treatment of at least 2 consolidation chemotherapies. We used *WT1* ProfileQuant™ kit (Ipsogen) for *WT1* monitoring. High *WT1* expression (>120 copies/10^4^ *ABL1*) after consolidation and at early period (3 months) after maintenance therapy significantly predicted subsequent relapse. All paired *PML-RARa* RQ-PCR were not detected except for one sample with early relapse. Patients with high *WT1* expression at 3 months after maintenance therapy (*n* = 40) showed a significantly higher relapse rate (30.5 vs. 6.9%, *P* < 0.001) and inferior disease free survival (62.8 vs. 91.4%, *P* < 0.001). Multivariate analysis revealed that high peak leukocyte counts at diagnosis (HR = 6.4, *P* < 0.001) and high *WT1* expression at 3 months after maintenance therapy (HR = 7.1, *P* < 0.001) were significant factors for prediction of relapse. Our data showed high post-remission *WT1* expression was a reliable marker for prediction of subsequent molecular relapse in APL. In this high-risk group, early intervention with ATRA ± ATO, anti-CD33 antibody therapy, and *WT1*-specific therapy may be used for relapse prevention.

**Trial registration:**

Clinical Research Information Service (CRIS), KCT0002079

**Electronic supplementary material:**

The online version of this article (doi:10.1186/s13045-017-0404-4) contains supplementary material, which is available to authorized users.

## Findings

In acute promyelocytic leukemia (APL), *PML-RARa* transcript is used as a marker for minimal residual disease (MRD), but the marker is not useful for pre-emptive management since its positivity directly indicates relapse. High Wilms’ tumor gene 1 (*WT1*) expression was related with subsequent relapse in acute myeloid leukemia, and Hecht et al. recently reported that high initial *WT1* expression was associated with more relapse in APL [1–3].

We confirmed APL by chromosome analysis and *PML-RARα* reverse transcriptase polymerase chain reaction (RT-PCR) method. All were treated with idarubicin (12 mg/m^2^, days 1, 3, 5, and 7) and all-trans retinoic acid (ATRA; 45 mg/m^2^/day) [4, 5]. After achievement of hematological complete remission (CR), all received three courses of consolidation—first, idarubicin (7 mg/m^2^, days 1–4); second, mitoxantrone (10 mg/m^2^, days 1–4); and third, idarubicin (12 mg/m^2^, day 1–2)—followed by 2-year maintenance using 6-mercaptopurine (50 mg/m^2^/day) plus ATRA [5–7]. The molecular studies were performed at diagnosis and 1 month after chemotherapy, and every 3 months after maintenance. Quantification of *PML-RARα* and *WT1* were performed using the real-time quantitative (RQ)-PCR methods (Real-Q *PML-RARα* quantification kit, Biosewoom, Korea, and *WT1* ProfileQuant™ kit, Ipsogen, France) presenting a similar sensitivity of 4.5 log.

We initially identified 142 APL patients from 2009 to 2014 but finally focused on 117 patients (median age 44 years old (range 19–70 years)) who underwent at least 2 cycles of consolidation after hematological CR. All patients were in complete molecular response (CMR) at the time of enrollment (Additional file 1: Figure S1, Table 1). Relapse was identified in 16 (13.7%) patients with a median duration of 22.8 months (range, 4.3–64.0). After median follow-up of 46.0 months (range, 14.7–86.3), 4-year cumulative incidence of relapse (CIR), non-relapse mortality (NRM), disease-free survival (DFS), and overall survival (OS) rates were 16.2, 1.2, 82.6, and 92.5%, respectively. We identified that high-risk Sanz-criteria, peak leukocyte count >40.0 × 10^9^/L, and *FLT3* mutation were predictive for relapse.

**Table 1:** Baseline characteristics of enrolled patients

Total *n* = 117	Number or median value
Age, median (range)	44 (19–70)
Gender, male	70 (59.8%)
Laboratory findings at diagnosis	
Leukocyte count (×10^9^/L)	2.68 (0.4–112.0)
Leukocytes count at peak (×10^9^/L)	16.6 (0.4–112.0)
Hemoglobin (g/dL)	8.9 (4.0–15.0)
Platelet (×10^9^/L)	33.0 (5.0–163.0)
Lactate dehydrogenase (LDH, U/L)	692 (250–4070)
Prothrombin time (PT, %)	63.0 (35.0–101.0%)
Partial thromboplastin time (aPTT, s)	28.0 (20–45)
Fibrinogen (mg/dL)	134.0 (31.0–500.0)
Antithrombin III (%)	94.0 (49.0–150.0)
D-dimer (mg/L)	17.0 (1.0–36.0)
Sanz criteria	
High	64 (54.7%)
Intermediate	19 (16.2%)
Low	34 (29.1%)
Karyotype	
Normal karyotype	5 (4.3%)
t(15;17) alone	79 (67.5%)
t(15;17) with 1 additional karyotype	20 (17.1%)
t(15;17) with ≥2 additional karyotype	13 (11.1%)
*PML-RARa* subtype	
BCR1	85 (72.6%)
BCR3	32 (27.4%)
*FLT3* mutation	
No *FLT3* mutation	86 (73.5%)
*FLT3*-ITD	25 (21.4%)
*FLT3*-TKD	6 (5.1%)
*WT1* (copies/10^4^ *ABL*), median (range)	
At diagnosis (*n* = 117)	18330 (20.0–236160.0)
Post-induction (*n* = 117)	63.9 (4.9–2360.0)
Post 1st consolidation (*n* = 117)	66.2 (1.1–2320.0)
Post 2nd consolidation (*n* = 117)	80.1 (1.3–2110.0)
Post 3rd consolidation (*n* = 117)	71.9 (10.8–808.0)
^a^Post-maintenance 3 months (*n* = 117)	70.0 (6.0–5520.0)
^a^Post-maintenance 1 year (*n* = 87)	57.5 (10.0–1630.0)
^a^Post-maintenance 2 year (*n* = 62)	54.4 (10.0–500.0)
At relapse (*n* = 16)	239.5 (77.1–34910.0)
Leukapheresis at initial treatment	20 (17.1%)
Differentiation syndrome	21 (17.9%)
Hematological complete response	
After induction	115 (98.3%)
After 2nd induction	2 (1.7%)
Complete molecular response (CMR)	
After induction	68 (58.1%)
After 2nd induction	2 (1.7%)
After 1st consolidation	42 (35.9%)
After 2nd consolidation	5 (4.3%)

*Abbreviation*: *BCR* breakpoint cluster region, *FLT3* Fms-related tyrosine kinase 3, *ITD* internal tandem duplication, *TKD* tyrosine kinase domain, *ABL* Abelson murine leukemia viral oncogene, *WT1*, Wilms tumor 1, *ATRA* all-trans retinoic acid, *AML* acute myeloid leukemia

^a^Post-maintenance indicates the time from starting maintenance therapy

We compared the level of *WT1* between relapsed and non-relapsed group during the course of treatment (Additional file 1: Figure S2) and identified that median *WT1* was significantly different at post 2nd consolidation (171.5 vs. 76.3, *P* = 0.049), at post 3rd consolidation (156.0 vs. 67.6, *P* = 0.013) and at 3 months post-maintenance (162.0 vs. 59.1, *P* = 0.002). We found that *WT1* post-maintenance 3 months was the most significant parameter for relapse prediction at the cutoff of ≥120.0 copies/10^4^ *ABL*.

**Fig. 1:**
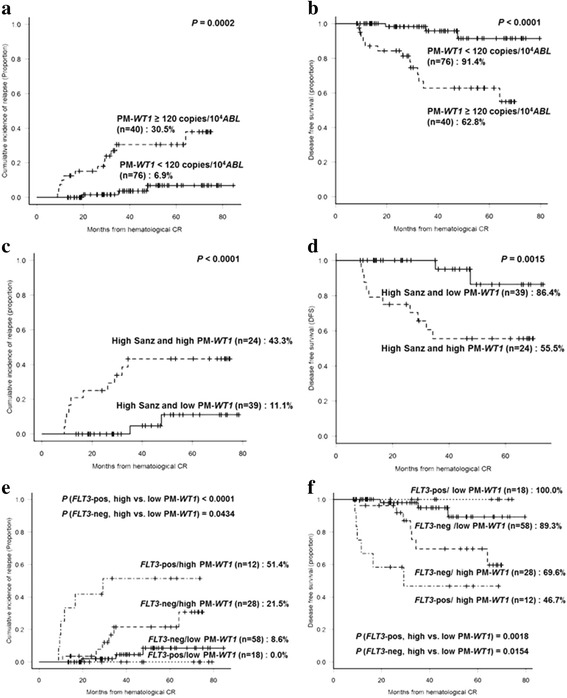
Treatment outcomes according to *WT1* expression level (<120 vs. ≥120 copies/10^4^ *ABL*) at 3 months post-maintenance (PM-*WT1*). **a** Four-year CIR rates. **b** Four-year DFS rates. **c**, **d** Four-year CIR and DFS rates according to *WT1* expression level in the high-risk subgroup. **e**, **f** Four-year CIR and DFS rates according to the status of PM-*WT1* and *FLT3*-ITD mutation

We calculated subsequent CIR and DFS rates in 116 patients after excluding 1 patient with early relapse. Patients with *WT1* post-maintenance 3 months higher than 120.0 copies/10^4^ *ABL* showed higher 4-year CIR (30.5 vs. 6.9%, *P* = 0.0002) and inferior 4-year DFS (62.8 vs. 91.4%, *P* < 0.0001) rates (Fig. 1a, b). Also in the high-risk subgroup, high *WT1* post-maintenance 3 months showed higher 4-year CIR (43.3 vs. 11.1%, *P* < 0.0001) and inferior 4-year DFS (55.5 vs. 86.4%, *P* = 0.0015) rates (Fig. 1c, d). In *FLT3* positive and negative subgroup, high *WT1* post-maintenance 3 months showed higher 4-year CIR (51.4 vs. 0.0%, *P* < 0.0001 and 21.5 vs. 8.6%, *P* = 0.0434) and inferior 4-year DFS (46.7 vs. 100.0%, *P* = 0.0018 and 69.6 vs. 89.3%, *P* = 0.0154) rates (Fig. 1e, f).

Multivariate analysis (Additional file 1: Table S1) revealed that 4-year CIR was significantly higher in patients with high peak leukocyte count (HR = 6.414; 95% CI, 2.1–19.3, *P* < 0.001) and high *WT1* post-maintenance 3 month (HR = 7.533; 95% CI, 2.3–24.8, *P* < 0.001), and 4-year DFS was significantly inferior in patients with high peak leukocyte count (HR = 5.275; 95% CI, 1.9–14.7, *P* = 0.001) and high *WT1* post-maintenance 3 month (HR = 8.241; 95% CI, 2.3–29.1, *P* = 0.001).

Unfortunately, our chemotherapy regimen was not differently specified for high-risk APL and the standard treatment of APL is now changed to a combination therapy using ATO. Therefore, current results may not be applicable in the treatment course using ATO and another validation is needed. Conclusively, high post-remission *WT1* expression is a reliable marker for prediction of subsequent relapse in APL patients treated with conventional chemotherapy. For patients with high-risk of relapse, early intervention using *WT1-*specific therapy may prevent relapse and improve survival outcomes [8, 9].

## Additional file

Additional file 1: Table S1. Multivariate analysis in APL patients with CMR. **Figure S1.** Consort diagram of enrolled patients in this study. Underlined patients were excluded in this study (n=25). Abbreviation: APL, acute promyelocytic leukemia; ATRA, all-trans retinoic acid; CR, complete remission, CMR, complete molecular response; *WT1*, Wilms tumor 1. **Figure S2.** Comparison of *PML-RARa* and *WT1* expression levels between relapsed and non-relapsed patients from diagnosis to relapse or 1 year after starting maintenance for non-relapsed patients. (DOCX 152 kb)
