# Supplementary material for: High WT1 expression is an early predictor for relapse in patients with acute promyelocytic leukemia in first remission with negative PML-RARa after anthracycline-based chemotherapy: a single-center cohort study
Source: J Hematol Oncol. 2017 Jan 23;10:30. doi: 10.1186/s13045-017-0404-4 (PMC5259829; doi:10.1186/s13045-017-0404-4)
Supplement: Additional file 1: Table S1. — Multivariate analysis in APL patients with CMR. Figure S1. Consort diagram of enrolled patients in this study. Underlined patients were excluded in this study (n=25). Abbreviation: APL, acute promyelocytic leukemia; ATRA, all-trans retinoic acid; CR, complete remission, CMR, complete molecular response; WT1, Wilms tumor 1. Figure S2. Comparison of PML-RARa and WT1 expression levels between relapsed and non-relapsed patients from diagnosis to relapse or 1 year after starting maintenance for non-relapsed patients. (DOCX 152 kb) [file 13045_2017_404_MOESM1_ESM.docx]

| **Table S1.** Multivariate analysis in APL patients with CMR. | | | | | | | | | | | | | |
| --- | --- | --- | --- | --- | --- | --- | --- | --- | --- | --- | --- | --- | --- |
| Variables |  | Relapse (CIR) | | | | |  | | Disease free survival (DFS) | | | | |
|  |  | Univariate | |  | Multivariate | | |  | Univariate | |  | Multivariate | |
|  |  | 4-year  CIR | *p* |  | HR  (95% CI) | *p* | |  | 4-year  DFS | *p* |  | HR  (95% CI) | *p* |
| Age at diagnosis |  |  |  |  |  |  | |  |  |  |  |  |  |
| < 45 years (n=60) |  | 20.0% | 0.626 |  |  |  | |  | 80.0% | 0.827 |  |  |  |
| ≥ 45 years (n=57) |  | 12.3% |  |  |  |  | |  | 85.2% |  |  |  |  |
| Additional chromosomes |  |  |  |  |  |  | |  |  |  |  |  |  |
| No aberrations (n=84) |  | 20.3% | 0.306 |  |  |  | |  | 77.9% | 0.247 |  |  |  |
| 1 or more aberrations (n=33) |  | 6.1% |  |  |  |  | |  | 93.9% |  |  |  |  |
| *FLT3* mutation |  |  |  |  |  |  | |  |  |  |  |  |  |
| No *FLT3* mutation (n=86) |  | 12.9% | 0.049* |  |  |  | |  | 85.5% | 0.063 |  |  |  |
| *FLT3*-ITD or –TKD (n=31) |  | 24.9% |  |  |  |  | |  | 75.1% |  |  |  |  |
| BCR subtype |  |  |  |  |  |  | |  |  |  |  |  |  |
| BCR1 (n=85) |  | 14.9% | 0.550 |  |  |  | |  | 83.4% | 0.650 |  |  |  |
| BCR3 (n=32) |  | 18.9% |  |  |  |  | |  | 81.1% |  |  |  |  |
| Sanz criteria |  |  |  |  |  |  | |  |  |  |  |  |  |
| Low to intermediate (n=53) |  | 4.7% | 0.022* |  |  |  | |  | 92.8% | 0.049* |  |  |  |
| High (n=64) |  | 25.6% |  |  |  |  | |  | 74.4% |  |  |  |  |
| Leukocyte count at peak |  |  |  |  |  |  | |  |  |  |  |  |  |
| < 40.0 x 10^9^/L (n=85) |  | 5.8% | <0.001* |  | 1 | <0.001* | |  | 92.5% | <0.001* |  | 1 | 0.001* |
| ≥ 40.0 x 10^9^/L (n=32) |  | 43.1% |  |  | 6.414  (2.1-19.3) |  |  |  | 56.9% |  |  | 5.275  (1.9-14.7) |  |
| Induction chemotherapy |  |  |  |  |  |  | |  |  |  |  |  |  |
| ATRA plus idarubicin (n=106) |  | 17.9% | 0.169 |  |  |  | |  | 80.7% | 0.164 |  |  |  |
| Others (n=11) |  | 0.0% |  |  |  |  | |  | 100% |  |  |  |  |
| CMR after induction chemotherapy |  |  |  |  |  |  | |  |  |  |  |  |  |
| CMR (n=68) |  | 14.3% | 0.552 |  |  |  | |  | 83.8% | 0.696 |  |  |  |
| No CMR (n=49) |  | 20.4% |  |  |  |  | |  | 79.6% |  |  |  |  |
| *WT1* at diagnosis |  |  |  |  |  |  | |  |  |  |  |  |  |
| < 1050 copies/10^4^*ABL* (n=31) |  | 26.5% | 0.092 |  |  |  | |  | 73.5% | 0.130 |  |  |  |
| ≥ 1050 copies/10^4^*ABL* (n=86) |  | 12.4% |  |  |  |  | |  | 85.8% |  |  |  |  |
| *WT1* at 3 months post-maintenance |  |  |  |  |  |  | |  |  |  |  |  |  |
| < 120 copies/10^4^*ABL* (n=76) |  | 6.9% | <0.001* |  | 1 | <0.001* | |  | 91.4% | <0.001* |  | 1 | 0.001* |
| ≥ 120 copies/10^4^*ABL* (n=40) |  | 30.5% |  |  | 7.533  (2.3-24.8) |  |  |  | 62.8% |  |  | 8.241  (2.3-29.1) |  |
| Abbreviation: CIR, cumulative incidence of relapse; HR, hazard ratio; *FLT3*, Fms-related tyrosine kinase 3; ITD, internal tandem duplication; TKD, tyrosine kinase domain; BCR, breakpoint cluster region; ATRA, all-trans retinoic acid; CMR, complete molecular response; *WT1*, Wilms tumor 1; *ABL*, Abelson murine leukemia viral oncogene; | | | | | | | | | | | | | |

| 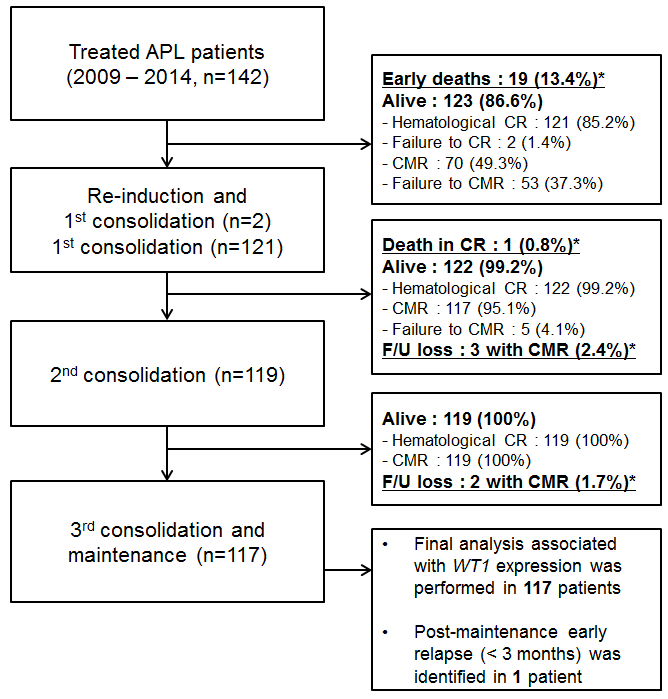 |
| --- |
| **Figure S1.** Consort diagram of enrolled patients in this study.  * Underlined patients were excluded in this study (n=25).  Abbreviation: APL, acute promyelocytic leukemia; ATRA, all-trans retinoic acid; CR, complete remission, CMR, complete molecular response; *WT1*, Wilms tumor 1; |

| 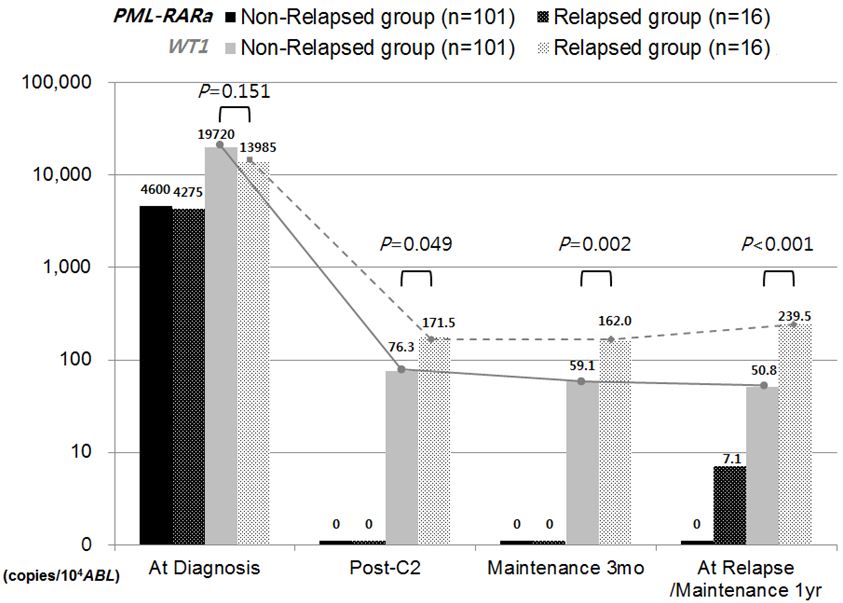 |
| --- |
| **Figure S2.** Comparison of *PML-RARa* and *WT1* expression levels between relapsed and non-relapsed patients from diagnosis to relapse or 1 year after starting maintenance for non-relapsed patients. |
